# Emergence of Multidrug-resistant Strain of *Vibrio cholerae* O1 in Bangladesh and Reversal of Their Susceptibility to Tetracycline after Two Years

**Published:** 2007-06

**Authors:** Abu S.G. Faruque, Khorshed Alam, Mohammad A. Malek, Mohammed G.Y. Khan, Sabeena Ahmed, Debasish Saha, Wasif A. Khan, Gopinath B. Nair, Mohammed A. Salam, Stephen P. Luby, David A. Sack

**Affiliations:** ICDDR,B GPO Box 128 Dhaka 1000 (Mohakhali, Dhaka 1212) Bangladesh

Sir,

Cholera is endemic and follows a distinct seasonality in Bangladesh (1). Early administration of rehydration therapy using appropriate oral or intravenous fluid(s) saves the lives of patients with cholera. Therapy with an effective antimicrobial agent significantly shortens the duration of diarrhoea and hospitalization, reduces the volume of watery stool and the requirement of maintenance fluids, and shortens the duration of faecal excretion of Vibrio cholerae reducing transmission of infection to other family members and nosocomial infections in the clinic setting (2).

Tetracycline and doxycycline (long-acting tetracycline) have long been the antibiotics of choice for treating severe cholera in Bangladesh and in other parts of the world, except for young children and pregnant women (1,2). Furazolidone, erythromycin, trimethoprim-sulphamethoxazole, and chloramphenicol have also been effective in treating severe cholera caused by strains of V. cholerae susceptible to these agents (3).

During October 2004–December 2005, 953 (565 Ogawa and 388 Inaba) and 344 (197 Ogawa and 147 Inaba) strains of V. cholerae O1 isolated from cholera cases admitted to the Dhaka Hospital (urban area) and Matlab Hospital (rural area) of ICDDR,B respectively were examined for susceptibility to tetracycline, erythromycin, trimethoprim-sulphamethoxazole, furazolidone, and ciprofloxacin. Antimicrobial susceptibility was performed using the disc-diffusion technique on Mueller-Hinton agar (Difco, Detroit, Michigan, USA) with commercial discs (Oxoid, UK) and appropriate control strains (4).

Overall, 525 (55%), 420 (44%), 944 (99%), 948 (100%), and no strains from Dhaka and 186 (54%), 165 (48%), 332 (97%), 343 (100%), and no strains from Matlab were resistant to tetracycline, erythromycin, trimethoprim-sulphamethoxazole, furazolidone, and ciprofloxacin respectively. Prior to October 2004, most strains were resistant to trimethoprim-sulphamethoxazole and furazolidone but were uniformly sensitive to tetracycline, erythromycin, and ciprofloxacin.

The first multidrug-resistant strains of V. cholerae (strains resistant to furazolidone, trimethoprim-sulphamethoxazole, tetracycline, and erythromycin) were observed in Matlab in October 2004 among the Ogawa serotype, and the isolation of such strains increased dramatically to reach 100% by February 2005. In Dhaka, the multidrug-resistant strains appeared one month later in November 2004 among both Ogawa (13%) and Inaba (5%) strains of V. cholerae, and by February 2005, all the Ogawa strains demonstrated the multidrug-resistant phenoytpe. By March 2005, nearly all the El Tor Ogawa isolates, 5 of 6 (83%) at the Matlab Hospital, and 43 of 45 (96%) at the Dhaka Hospital were multidrug-resistant. The figure shows the resistance patterns of V. cholerae in Dhaka and Matlab by month and site.

**Fig.**
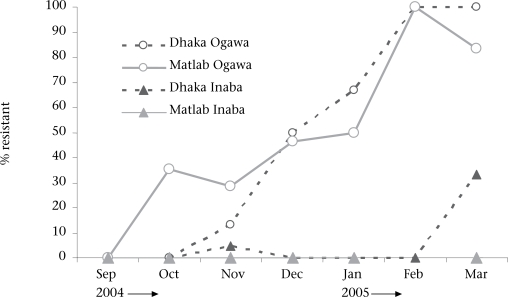
Resistance patterns of *V. cholerae* O1 in Dhaka and Matlab by month and site

Based on the multidrug-resistant phenotype, it appeared that there were two or more clones of V. cholerae O1 in circulation at different time points, and we are in the process of understanding the clonality by subjecting representative strains to molecular typing analysis. We determined antimicrobial susceptibility using both disc-diffusion technique and E-test method.

In the absence of a reference zone size for V. cholerae resistance to erythromycin and azithromycin, we used the zone size for other organisms to determine the susceptibility (zone of inhibition ≥23 mm for erythromycin and ≥18 mm for azithromycin) of the V. cholerae strains to these drugs. Seventeen of the 35 (49%) V. cholerae strains were resistant to both erythromycin and azithromycin, while the remaining 18 (51%) strains were susceptible to both erythromycin and azithromycin. The strains resistant to erythromycin exhibited minimum inhibition concentrations (MICs) of 8 to 32 μg/mL, while the MIC of those susceptible to the drug ranged from 0.25 to 1 μg/mL. The MIC of the strains resistant to azithromycin ranged from 0.75 to 3 μg/mL, while the MIC of those susceptible to the drug was from 0.047 to 0.125 μg/mL. These findings suggest that resistance to erythromycin could be a marker for resistance to azithromycin. Similarly, resistance to tetracycline is considered a proxy indicator for resistance to doxycycline (5). We have, for the first time, encountered this unique, multidrug-resistant pattern, including resistance to erythromycin, among V. cholerae O1 in Bangladesh.

Throughout the period of this investigation, all isolates from Matlab and Dhaka were susceptible to ciprofloxacin. The MIC for ciprofloxacin was determined using E-test (AB-BIODISK, Sweden) with the zone size interpretive criteria for susceptibility corresponding to 0.06 μg/mL (5). We noted a consistent increase in the median MIC of V. cholerae O1 strains isolated at the Dhaka Hospital over the years: 0.003 μg/mL in 1994, 0.023 μg/mL in 2001, and 0.38 to 0.5 μg/mL in 2005 (6,7). In a recent study in adults infected with V. cholerae O1, we observed clinical success (resolution of diarrhoea by 48 hours of initiation of therapy) rate of 68% with 500-mg 12-hourly dose of ciprofloxacin for three days—a rate substantially lower than that observed with a single one-gramme dose of the drug in an earlier study (8).

The findings are disturbing since a further increase in the MIC may render ciprofloxacin ineffective in the management of cholera caused by such multidrug-resistant strains of V. cholerae O1. Further studies are being conducted at ICDDR,B to determine the mechanism of resistance of these multidrug-resistant strains to ciprofloxacin.

In August 2006, we observed a re-emergence of the Inaba serotype and a sharp reduction in the isolation rate of the Ogawa serotype. In Bangladesh, such changes in serotypes and the appearance of short-lasting, multidrug-resistance, have also been noted in the past (9,10,11). The proportion of V. cholerae strains resistant to tetracycline decreased substantially (from 75% in January 2006 to 10% in August 2006). Resistance to tetracycline persisted for nearly two years, a finding that we have not observed earlier (12,13).

The current situation clearly demonstrates the need to monitor MIC in areas where cholera is endemic to assess the clinical efficacy of ciprofloxacin in the treatment of cholera. There is also a need to identify effective alterative antimicrobials for the management of cholera caused by such strains. Through this communication, we would also like to alert our neighbouring countries and the region of circulation of this new multidrug-resistant strain of V. cholerae O1.

## References

[1] Sack DA, Sack RB, Nair GB, Siddique AK (2004). Cholera. Lancet.

[2] Greenough WB, Gordon RS, Rosenberg IS, Davies BI, Benenson AS (1964). Tetracycline in the treatment of cholera. Lancet.

[3] World Health Organization (1993). Guidelines for cholera control.

[4] National Committee for Clinical Laboratory Standards (1990). Performance standards for antimicrobial disc susceptibility tests. Approved standard M2-A5.

[5] Koplan JP, Hughes JM, Cohen ML, Samba EM, Kabore AB, Bopp CA (1999). Laboratory methods for the diagnosis of epidemic dysentery and cholera.

[6] Khan WA, Bennish ML, Seas C, Khan EH, Ronan A, Dhar U (1996). Randomized controlled comparison of single-dose ciprofloxacin and doxycycline for cholera caused by *Vibrio cholerae* O1 or O139. Lancet.

[7] Saha D, Khan WA, Karim MM, Chowdhury HR, Salam MA, Bennish ML (2005). Single-dose ciprofloxacin versus 12-dose erythromycin for childhood cholera: a randomised controlled trial. Lancet.

[8] Salam MA, Saha D, Karim MM, Khan WA, Ahmed S (2005). The quest for new drug in the treatment of cholera in adults: evaluation of efficacy of rifaximin in a randomized, controlled clinical trial (abstract). 40th Joint Meeting of the US-Japan Cholera and Other Bacterial Enteric Infections Panel.

[9] Siddique AK, Zaman K, Majumder Y, Islam Q, Bashir I, Mutsuddy P (1989). Simultaneous outbreaks of contrasting drug resistant classic and El Tor Vibrio cholerae O1 in Bangladesh. Lancet.

[10] Urassa WK, Mhando YB, Mhalu FS, Mjonga SJ (2000). Antimicrobial susceptibility pattern of Vibrio cholerae O1 strains during two cholera outbreaks in Dar es Salaam, Tanzania. East Afr Med J.

[11] Zachariah R, Harries AD, Arendt V, Nchingula D, Chimtulo F, Courteille O (2002). Characteristics of a cholera outbreak, patterns of Vibrio cholerae and antibiotic susceptibility testing in rural Malawi. Trans R Soc Trop Med Hyg.

[12] Glass RI, Huq I, Alim ARMA, Yunus M (1980). Emergence of multiply antibiotic-resistant Vibrio cholerae in Bangladesh. J Infect Dis.

[13] Glass RI, Huq MI, Lee V, Threlfall EJ, Khan MR, Alim ARMA (1983). Plasmid-borne multiple drug resistance in Vibrio cholerae serogroup O1, biotype El Tor: evidence for a point-source outbreak in Bangladesh. J Infect Dis.

